# Blood perfusion in osteomyelitis studied with [^15^O]water PET in a juvenile porcine model

**DOI:** 10.1186/s13550-016-0251-2

**Published:** 2017-01-14

**Authors:** Lars Jødal, Ole L. Nielsen, Pia Afzelius, Aage K. O. Alstrup, Søren B. Hansen

**Affiliations:** 1 Department of Veterinary Disease Biology, University of Copenhagen, Copenhagen, Denmark; 2 Department of Nuclear Medicine and PET Centre, Aarhus University Hospital, Aarhus, Denmark; 3 Department of Nuclear Medicine, Aalborg University Hospital, P.O. Box 365, 9100 Aalborg, Denmark; 4 Department of Diagnostic Imaging, North Zealand Hospital, Hillerød, Copenhagen University Hospital, Copenhagen, Denmark

**Keywords:** Osteomyelitis, Perfusion, Positron emission tomography, [^15^O]water, Swine, Bone infection

## Abstract

**Background:**

Osteomyelitis is a serious disease which can be difficult to treat despite properly instituted antibiotic therapy. This appears to be related at least partly to degraded vascularisation in the osteomyelitic (OM) lesions. Studies of perfusion in OM bones are, however, few and not quantitative. Quantitative assessment of perfusion could aid in the selection of therapy. A non-invasive, quantitative way to study perfusion is dynamic [^15^O]water positron emission tomography (PET). We aim to demonstrate that the method can be used for measuring perfusion in OM lesions and hypothesize that perfusion will be less elevated in OM lesions than in soft tissue (ST) infection. The study comprised 11 juvenile pigs with haematogenous osteomyelitis induced by injection of *Staphylococcus aureus* into the right femoral artery 1 week before scanning (in one pig, 2 weeks). The pigs were dynamically PET scanned with [^15^O]water to quantify blood perfusion. OM lesions (*N* = 17) in long bones were studied, using the left limb as reference. ST lesions (*N* = 8) were studied similarly.

**Results:**

Perfusion was quantitatively determined. Perfusion was elevated by a factor 1.5 in OM lesions and by a factor 6 in ST lesions.

**Conclusions:**

Blood perfusion was successfully determined in pathological subacute OM lesions; average perfusion was increased compared to that in a healthy bone, but as hypothesized, the increase was less than in ST lesions, indicating that the infected bone has less perfusion reserve than the infected soft tissue.

**Electronic supplementary material:**

The online version of this article (doi:10.1186/s13550-016-0251-2) contains supplementary material, which is available to authorized users.

## Background

Osteomyelitis destroys bone tissue, affecting blood perfusion of the bone in the process [1]. Paediatric haematogenous osteomyelitis typically involves long bones of the extremities, most frequently the metaphyseal region, apparently originating in the capillary loop structure vascularizing the bone’s growth region [2, 3]. Osteomyelitis is often hard to treat with antibiotics, which can be related to the degradation of the vascular structure in the osteomyelitic (OM) area [1]. Studies of blood perfusion in OM lesions are, however, scarce, probably because of the difficulties involved with measuring perfusion within the bone.

Colour Doppler ultrasound has been used to measure blood flow in osteomyelitis [4, 5]; both of these studies were on children with recent symptoms (2–15 days in one study, 2–10 days in the other), and both found elevated blood flow within or around the involved periosteum. Ultrasound, however, cannot evaluate the bone marrow [6]. Wannfors and Gazelius [7] applied laser Doppler flowmetry (LDF) directly to the jaw bones affected by chronic osteomyelitis (duration 1–26 years), finding bone blood flow to be decreased (relative to the healthy side of the jaw) in patients with low clinical activity of the disease, while being increased in patients with high clinical activity of the disease; furthermore, blood flow was found to be increased in the initial subacute lesions. Müller et al. [8] combined contrast-enhanced ultrasound with static [^18^F]NaF positron emission tomography/computed tomography (PET/CT) to assess bone microcirculation in ten patients (only three with osteomyelitis) undergoing mandibular reconstruction. The study did not report quantitative perfusion, though, but found PET/CT valuable as a tool in the assessment. Two recent papers describe experiences with dynamic PET/CT, using [^18^F]NaF [9] or [^18^F]FDG [10] as tracer; both studies reported increased standardized uptake values (SUV) in the volume of interest (VOI) positioned at the site of chronic OM infection.

None of the above studies report quantitative results on bone perfusion in osteomyelitis, at best relative values like SUV. Quantitative results will give more precise results and could allow the physician to select the best and most individualized therapy.

Quantitative in vivo measurement of blood perfusion in the bone is feasible with dynamic [^15^O]water (H_2_^15^O) PET/CT, as demonstrated in several studies [11–16]. But to our knowledge, [^15^O]water PET/CT has not been applied before to osteomyelitis.

We set out to demonstrate that [^15^O]water PET/CT is useful for determining perfusion in OM lesions in a juvenile porcine animal model, which is representative for haematogenous osteomyelitis in children [17].

Acute and subacute infections usually involve increased perfusion of the tissue, which helps the body’s transport of white blood cells, antibodies, complement and other soluble substances to the infected area. Based on the destructive nature of the OM disease, we hypothesized that the perfusion increase will be less in OM lesions than in soft tissue (ST) lesions. Such lack of increase could also be part of the explanation why osteomyelitis can be hard to treat with antibiotics [1]. To test the hypothesis, we measured perfusion in our juvenile porcine model and compared the perfusion increase in OM and ST lesions.

## Methods

The present study reports new [^15^O]water PET data from an osteomyelitis multi-tracer project on infection scanning [18–20]. The study includes all the pigs reported in these studies and two more, for a total of 11 pigs; general data on the pigs are given in Table 1.

**Table 1:** Data for pigs

Pig no.^a^	Weight (kg)^b^	Days from inoculation until scanning	Activity injected for [^15^O]water PET (MBq)
1	40	7	500
2	40	7	1000
3^c^	39	7	1000
4	42	7	850
5	20	14	950
6	22	7	832
7	23	7	956
8	21	8	1000
9	23	8	1000
10	22.5	7	1000
11	19	7	500

^a^Consecutive numbering of PET scanned pigs. Physiological data for pigs no. 1–4 have been described in [20] and for pigs no. 6–10 in [19]. Pig no. 5 was scanned 14 days after inoculation in an attempt to study more chronic infections, but this extended infection period was difficult to pursue because the pigs reached the approved humane endpoint

^b^Body weight was changed from approx 40 to 20 kg as part of the refinement of the porcine osteomyelitis model [40]. Shortly, our refinement study showed that fewer pigs had to be killed due to humane endpoints when smaller pigs were used and when they were treated with penicillin just after onset of the first clinical symptoms of lameness

^c^Infection did not catch in pig no. 3, but the pig is retained for the comparison of right and left hind limbs in uninfected tissue (see main text)

Briefly, OM lesions were induced in the right hind limb of juvenile Danish Yorkshire-Landrace cross-breed female pigs by intra-arterial injection of *Staphylococcus aureus* (porcine strain S54F9). Injection was into the right femoral artery according to the technique developed by Johansen et al. [21]. This protocol allows selective infection in one hind limb, leaving the other hind limb as a healthy control. Osteomyelitis was allowed to develop for 1 week (2 weeks in one pig, see note to Table 1), after which the pigs were scanned and then euthanized. The protocol was approved by the Danish Animal Experimentation Board, journal no. 2012-15-2934-00123.

### Water scans

PET scanning was performed on a Biograph TruePoint 64 PET/CT (Siemens, Erlangen, Germany). A bolus of 500–1000 MBq of [^15^O]water was injected in a jugular vein at the start of PET scan. The PET scan lasted 5 min and comprised the following time frames: 12 × 5, 6 × 10 and 9 × 20 s. The scanning region covered 21 cm in the axial direction, positioned over the pelvic region and hind limbs.

PET data were corrected for photon attenuation and scatter and reconstructed using attenuation-weighted ordered subset expectation maximum (OSEM) with resolution recovery (TrueX, Siemens). Reconstruction parameters were 6 iterations, 21 subsets, 336 × 336 matrix in 109 slices, voxel size 2 × 2 × 2 mm^3^ and a 2-mm Gaussian filter. The spatial resolution of the images was about 4 mm. Activity concentrations were decay-corrected back to the start of the PET recording.

During [^15^O]water PET, blood was sampled from the carotid artery using an automatic blood sampler (Allogg AB, Sweden) with readout every second. Only events registered within an energy window centred at the 1022 keV (2 × 511 keV) peak were used in order to suppress background counts of 511 keV photons from activity outside the counter (i.e. in the pig being scanned). This setup also excluded possible contamination from non-PET radiotracers in the blood samples (which could happen with ^111^In [22]). The multi-tracer study included more than one PET tracer in the same pig, but the [^15^O]water scans were performed first or at least five half-lives after administration of prior radiotracer (^11^C tracers, *T*_½_ = 20 min); neither images nor blood data showed indications of signal contamination.

### VOIs

Comparisons of right and left hind limb in regions without signs of infection were performed in each pig by drawing volumes of interest (VOIs) in the femoral medullar canal and in the thigh muscles. Each femoral medulla VOI was combined from a series of 2D regions of interest (ROIs), while the muscle VOIs were relatively large spheres (volumes 40–75 cm^3^) including neither bone nor skin.

OM and ST infectious lesions were identified and characterized from a combination of CT, gross pathology, histopathology and microbiology results; for details, see Refs. [18–20]. Briefly, all bone lesions showed osteolysis on CT; all pigs were necropsied for verification of the presence of lesions (both OM and ST). Except for pig no. 6, *S. aureus* was re-isolated from one or two lesions in each of the pigs; pig no. 6 was found positive for *S. aureus* by immunohistochemistry in both of the lesions tested. Histopathology was performed on selected lesions only.

Only lesions (OM and ST) visible on CT were considered in the present study. OM VOIs were drawn at the sites of osteolysis, attempting to avoid visible sequestra. ST VOIs in non-abscess regions were drawn at the centre of the swelled region; for abscesses, a representative position at the capsule was sought—see the “Results” section for more details. For reproducibility, the VOIs were in most cases drawn as spheres, with volumes in the range 1 to 5 cm^3^. Unless otherwise noted, VOI drawing was based on CT alone.

For each studied limb lesion, a VOI was drawn at the site of the lesion, and a similar VOI was drawn in the anatomically corresponding position in the non-infected (i.e. left) limb.

The modest spatial resolution of PET was a limitation for lesions in the pedal bones, where the small OM lesions could not reliably be separated from nearby soft tissue lesions; pedal OM lesions (pigs no. 1, 4, 9–11) were therefore excluded from the study. The pedal ST lesions were larger and were included.

VOI drawing was performed in the software Carimas version 2.9 (Turku PET Center, www.turkupetcentre.fi/carimas/) [23].

### Robustness of OM lesion VOI drawing

Optimal positioning of the OM lesion VOIs was sometimes difficult. An initial test was therefore performed on a sample of OM lesion VOIs. The sample comprised both proximal and distal lesions from both femur and tibia, in total eight OM lesions (more precisely, all long bone lesions from pigs no. 1, 5 and 6 and the distal tibial lesions from pigs no. 7 and 8, see Table 3 in the “Results” section).

The test went as follows: Six copies of each VOI were made and moved two voxels (4 mm) lateral, medial, anterior, posterior, dorsal or ventral to the original lesion VOI. Perfusion (*K*_1_ value, see below) was calculated for both original and surrounding (moved) VOIs. This was done for both lesion VOIs (right limb) and for the corresponding VOIs in the non-infected side (left limb).

Because this was an initial test to support the VOI drawing in general, the results are reported here, not in the “Results” section. Thus, for all the OM lesions, it was found that the original VOI had higher perfusion than the average value from its surrounding VOIs. Comparing with the six surrounding VOIs individually (unaveraged), the original VOI had the highest perfusion in ~85% of comparisons. For the VOIs in the non-infected positions, differences were non-systematic, as might be expected for positions in tissue without a specific (lesion) centre.

The findings were taken as a confirmation that the lesion VOIs were correctly focused on a position differing systematically from the surroundings (i.e. representing perfusion in the OM lesion), and that, the VOIs in the non-infected positions were representative for these more homogeneous positions.

### Kinetic modelling

Kinetic modelling was performed on the dynamic water PET data with a one-tissue compartment model (1TCM), using the blood data as input function [24, 25]. Water is highly diffusible, and extraction fraction (EF) will be close to 100%; for cardiac tissue in dogs, Bergmann et al. [26] found mean EF for [^15^O]water to be 96 ± 5% for perfusion up to 100 mL/min/100 g. Assuming full extraction, uptake will be all incoming [^15^O]water, i.e. *K*_1_ will directly express perfusion (for illustration with brief explanation, cf. Additional file 1: Figure S1).

Comparison of VOI data and input blood data (input function) showed that bolus of the input function appeared slightly later than the bolus in the VOI data. This time difference could be explained as the combined effect of delay in the tubing of the blood sampling and possible physiological offset between the point of blood sampling and the organs. Based on preliminary fitting with time delay treated as a free fitting parameter, the time difference was determined to average −5 s. The results reported here are based on a fixed −5 s offset of the input function.

Kinetic modelling was performed on data from VOIs, using the fit_h2o program developed by Oikonen at Turku PET Center [27, 28] (the Carimas software can also be used for such calculations, but the fit_h2o program is better suited for batch scripts).

Parametric images of the perfusion were calculated on a voxel-per-voxel basis, using the Carimas software. To reduce noise, the parametric images were calculated from PET images smoothed with a 3D Gauss function of full width at half maximum (FWHM) 2.5 mm. These parametric images were used for illustrative purposes (see images in the “Results” section), not for quantitative reporting; for the few soft tissue lesions where VOI drawing was partly guided by PET images (see the “Results” section), the parametric images were part of the guiding.

### Statistics

Perfusion (*F* = *K*_1_) within the left and right limb was compared in paired VOIs. For the comparison, perfusion differences were calculated as simple difference and logarithmic difference:

$$ \mathrm{simple}\ \mathrm{difference} = {F}_{\mathrm{right}}\ \hbox{--}\ {F}_{\mathrm{left}} $$

$$ \mathrm{logarithmic}\ \mathrm{difference} = \ln \left({F}_{\mathrm{right}}\right)\ \hbox{--}\ \ln \left({F}_{\mathrm{left}}\right) = \ln \left({F}_{\mathrm{right}}/{F}_{\mathrm{left}}\right) $$

Simple differences are most representative if the difference level is independent of the perfusion level. Logarithmic differences will be more representative if the right/left ratio (rather than the simple difference) is independent of the perfusion level, in which case the average side difference will grow proportionally with the perfusion level.

The statistical significance of differences (both simple and logarithmic) was tested with Student’s paired *t* test for normally distributed data and with Wilcoxon’s signed rank test for non-normally distributed data. Comparison between groups (OM vs. ST lesions) was done with unpaired *t* test when both groups had normal distribution and with Mann-Whitney *U* test when not. Normality of data was tested with the Shapiro-Wilk *W* test. Statistical tests were performed with StatsDirect version 3.0.171 (www.statsdirect.com). All tests used two-sided *p* values, and *p* < 0.05 was considered statistically significant.

For the OM and ST lesions, Bland-Altman plots [29] were used, allowing graphical evaluation of the homogeneity of differences.

## Results

### Perfusion in femoral medullar canal and thigh muscle

**Fig. 1:**
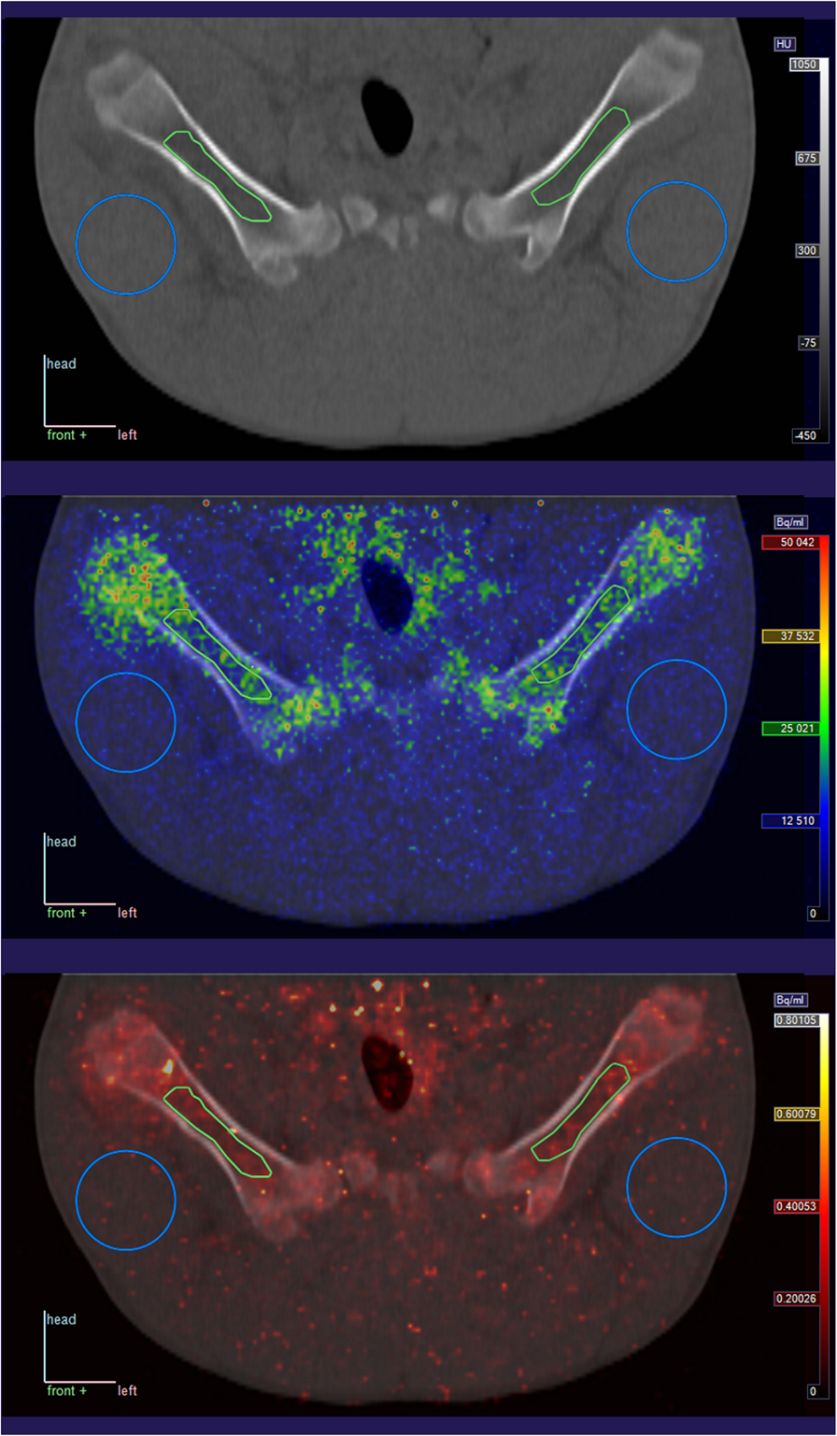
Example of scanning (pig no. 1). *Top*: CT image. *Middle*: Fusion of CT and [^15^O]water PET images (averaged over the 5 min scanning time). *Bottom*: Fusion of CT and parametric image of the *K*_1_ parameter, i.e. the perfusion, with colour scale from 0 to 80 mL/min/100 cm^3^. In the images, a section is shown of the 3D VOIs drawn in the femoral medullar canal and in the thigh muscles. In this example, the positioning of the pig was very symmetric; in cases where pigs were slightly tilted to one side or had variation in how much the limbs were flexed, VOIs were drawn to correspond anatomically, not necessarily centred within the same slice

This comparison was made in all 11 pigs, in order to compare the right and left hind limb in uninfected locations. An example of VOI drawing in the marrow and muscle is shown in Fig. 1. Perfusion data are shown graphically in Additional file 1: Figures S2 and S3.

Summary statistics are given in Table 2 (detailed statistics in Additional file 1: Table S1). In the femoral medullar canal, no significant side difference was found. In the thigh muscles, the right hind limbs had on average 11% less perfusion than the left hind limbs, which was statistically significant.

**Table 2:** Summary statistics on perfusion in right (infected) and left (non-infected) hind limbs

VOI location	Number of pairs	Mean ± SD, mL/min/100 cm^3^		Ratio from logarithmic^a^	Right vs. left*
		Right	Left		
Medullary canal	11	21 ± 11	21 ± 14	1.06	*p* > 0.1 (NS)
Thigh muscle	11	3.8 ± 0.9	4.3 ± 1.3	0.89	*p* < 0.02
OM lesions^b^	17	39 ± 13	26 ± 7	1.46	*p* < 0.002
ST lesions^c^	8	41 ± 13	7.0 ± 5.4	6.1	*p* < 0.01

*NS* not significant

*Combining *p* values from simple and logarithmic differences, e.g. *p* < 0.01 means that the *p* value was below 0.01 for *both* simple and logarithmic differences. For detailed comparison results, see Additional file 1: Table S1

^a^Representing result from logarithmic differences; e.g. for medullary canal, the mean value of logarithmic differences was 0.058, giving ratio = exp (0.058) = 1.06

^b^Only long bones (not patella), primary data in Table 3

^c^Primary data in Table 4

### Perfusion of OM lesions

**Fig. 2:**
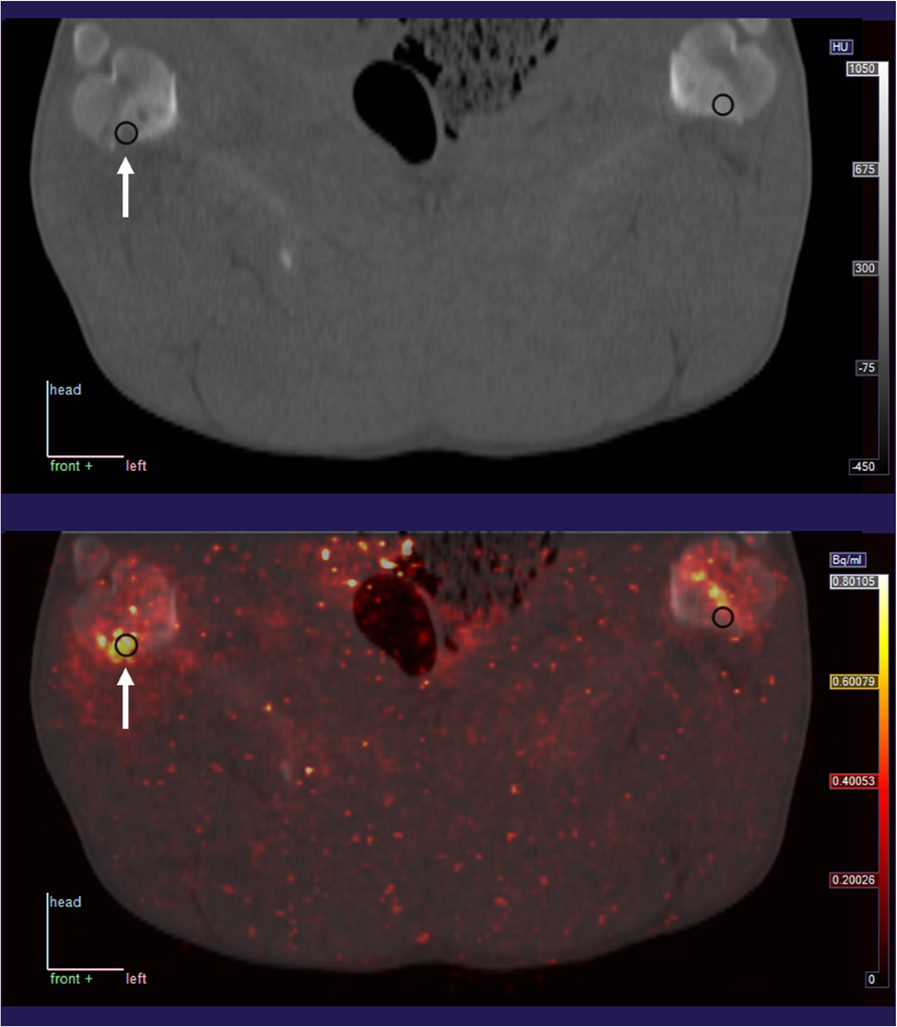
Example of lesion (*arrow*) and spherical VOIs drawn at the lesion and at the corresponding position in the non-infected limb. The *upper* image is CT, and the *lower* image shows CT fused with parametric image of perfusion. This is the same pig and same colour scale as in Fig. 1

Pig no. 3 was without infection on the limbs, and the only OM lesions in pigs no. 4 and 11 were small pedal lesions, for which a bone VOI could not be reliably drawn (cf. Methods section). An example of the VOI drawing is given in Fig. 2. Results for perfusion in the included OM lesions and corresponding positions in the non-infected (left) limbs are presented in Table 3.

**Table 3:** Perfusion of OM lesions in the long bones (right hind limb) and of the corresponding non-infected positions (left hind limb). Unless otherwise noted, the VOIs were spherical

Region	Pig no.	Perfusion, mL/min/100 cm^3a^			Right/left
		Right	Left	Right–left	
Proximal femur^b^	1	29	26	3	1.1
Distal femur	1	41	20	21	2.1
Distal femur^b, c^	5	52	27	25	1.9
Distal femur	6	53	20	33	2.6
Distal femur^b, c^	7	56	24	32	2.3
Distal femur	8	24	22	2	1.1
Distal femur	9	27	18	9	1.5
Distal femur	10	25	34	−9	0.7
Proximal tibia	1	34	33	1	1.0
Proximal tibia^c^	6	35	31	4	1.1
Proximal tibia^b^	7	36	35	2	1.1
Proximal tibia^b^	8	42	29	12	1.4
Proximal tibia^b^	9	26	14	12	1.8
Proximal tibia^b^	10	35	38	−3	0.9
Distal tibia^b, c^	7	43	27	16	1.6
Distal tibia^b^	8	70	33	37	2.1
Distal tibia	10	36	17	19	2.1
Patella^d^	2	23	37	−14	0.6

^a^Conversion to unit mL/min/100 g may be performed by division with tissue density. According to Woodard et al. [41], tissue density is 1.05 g/cm^3^ for the skeletal muscle, 1.03 g/cm^3^ for the red marrow, 0.98 g/cm^3^ for the yellow marrow and 1.92 g/cm^3^ for the cortical bone

^b^The lesion contained sequester or developing sequester

^c^Custom-drawn or elliptical VOI for irregular lesion; corresponding VOI in left limb was spherical

^d^The patella was necrotic and left out of the statistical analysis; see main text

The histological examination of the patella lesion in pig no. 2 indicated that it was necrotic. As such, it was considered an outlier for the determination of perfusion and excluded, leaving 17 OM lesions for statistical comparisons.

Summary statistics for the OM lesions are given in Table 2. Perfusion in the OM lesions was on average elevated by 39 − 26 = 13 mL/min/100 cm^3^ or a factor of about 1.5.

### ST lesions

**Fig. 3:**
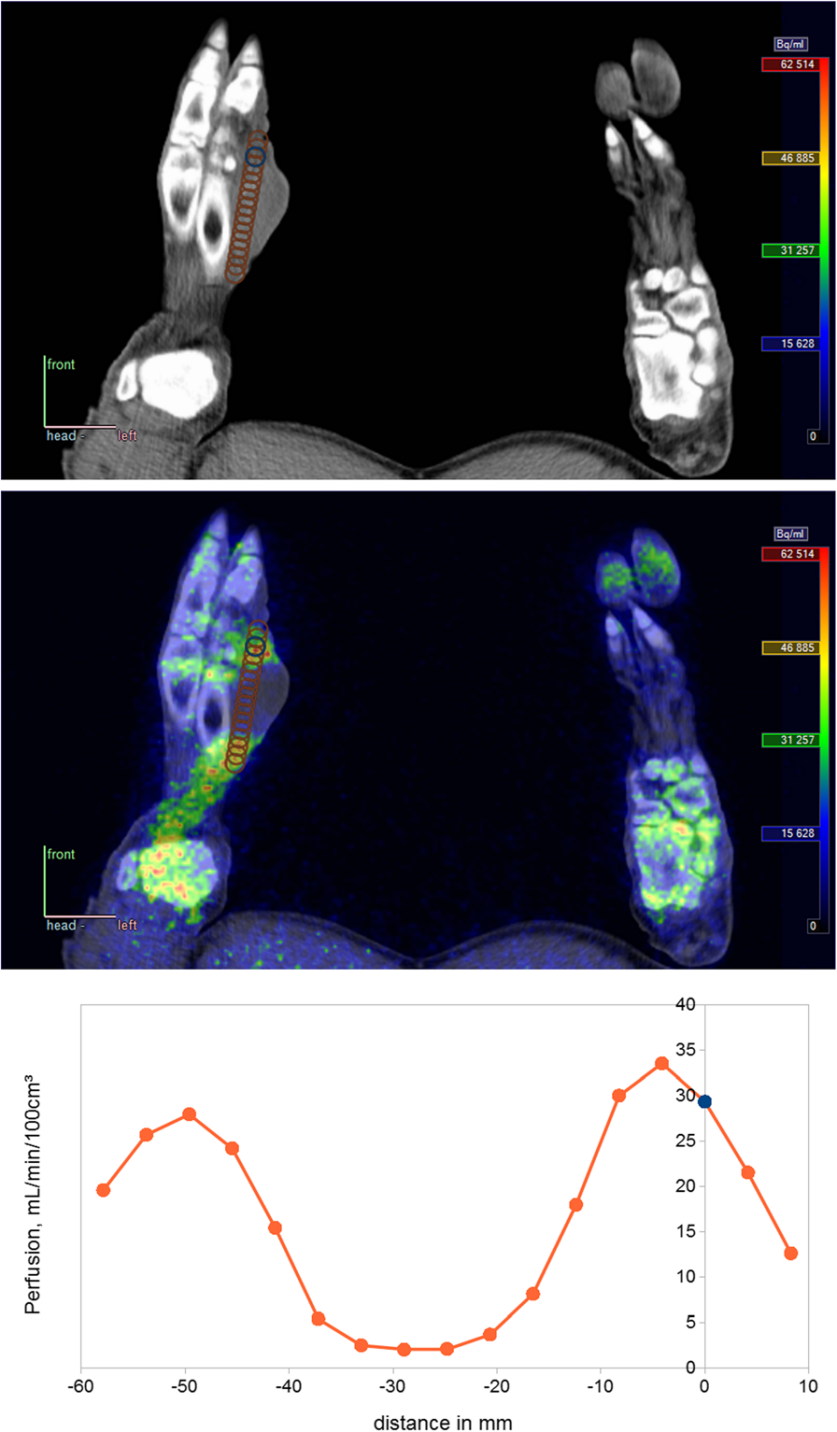
Abscess at metatarsus II in pig no. 4. *Top*: CT image with chosen VOI position reported in Table 4, determined from CT with guidance from [^15^O]water PET image. *Middle*: Fused PET/CT image with a profile of VOIs at vertical spacing of 2 voxels. *Bottom*: Perfusion profile from these VOIs. The chosen VOI corresponds to distance 0 mm and turns out not to be maximum value, but judged from other side of the profile, the value appears to be typical for the non-necrotic part of the abscess

Peri-osseous ST abscesses developed in several of the pigs and included abscesses near the pedal OM lesions. Unlike the OM lesions, these ST lesions were large enough to be included. In one pig, an abscess also developed at the injection site. Some of these abscesses had very inhomogeneous perfusion due to necrotic centres, which made VOI drawing a challenge in order to represent a “typical” perfusion of the lesion. In these cases, VOIs were drawn on the non-necrotic capsule of the abscess, still based on CT images, but with guidance from the [^15^O]water PET images to avoid the necrotic centre. The most pronounced case is presented in Fig. 3.

The ST lesion results are presented in Table 4 (*n* = 8 lesions in seven pigs). Summary statistics are given in Table 2. Perfusion in the ST lesions was on average elevated by 41 − 7.0 = 34 mL/min/100 cm^3^ or a factor of about 6.

**Table 4:** Perfusion of ST lesions (right hind limb) and corresponding non-infected positions (left hind limb). Except for the abscess at injection site in pig no. 4, these soft tissue lesions were next to bone lesions

Region	Pig no.	Perfusion, mL/min/100 cm^3a^			Right/left
		Right	Left	Right–left	
Abscess at metatarsus III	1	21	5	16	4.0
Phlegmon at patella	2	62	5	56	11.3
Capsule of abscess at injection site^b^	4	39	4	35	10.0
Capsule of abscess at metatarsus II^c^	4	29	8	21	3.6
Abscess at distal tibia	8	54	14	40	3.8
Abscess at calcaneus^b^	9	38	5	33	7.9
Abscess plantar to phalanx IV	10	40	10	30	4.1
Abscess at calcaneus	11	44	4	39	10.1

^a^Regarding unit for perfusion, see footnote in Table 3

^b^VOI position to some extent guided by PET image

^c^VOI position guided by PET image. See also Fig. 3

### OM lesions vs. ST lesions

**Fig. 4:**
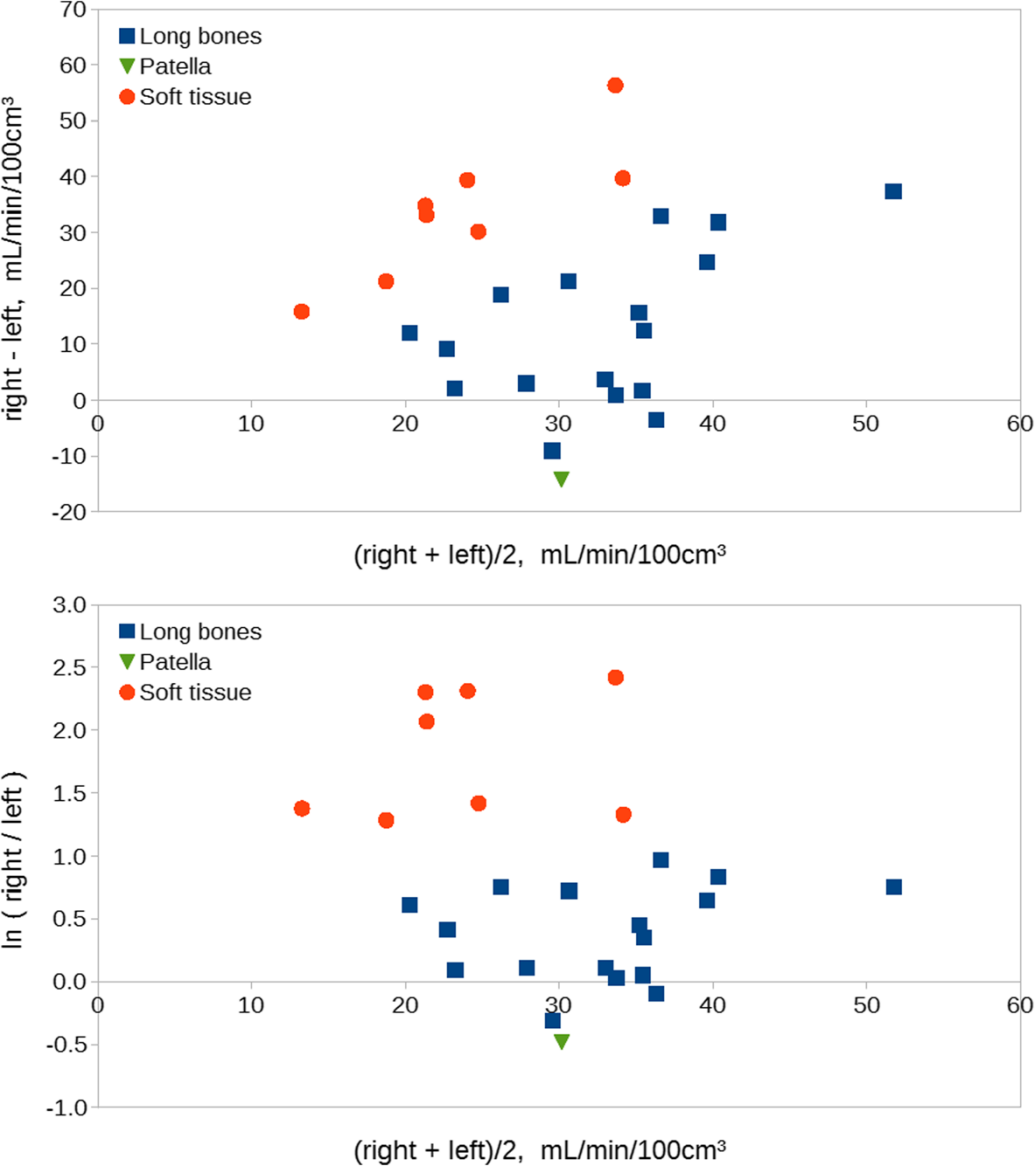
Bland-Altman plots for perfusion in long bone and soft tissue lesions. *Upper*: simple differences. *Lower*: logarithmic differences (a difference ∆ corresponds to a factor of exp(∆), e.g. exp(1.1) ≈ 3 and exp(2.3) ≈ 10). Underlying data are presented in Table 3. Note the more homogeneous spread of OM and ST in the logarithmic plot

The perfusion data given above are presented as Bland-Altman plots in Fig. 4. The differences represent the increased perfusion in the infected sites. The logarithmic differences appear more independent of perfusion (regarding both level and spread of the differences) than does the simple differences. This favours interpretations based on the logarithmic differences, e.g. as ratios.

Perfusion increase was higher in ST lesions than in OM lesions (*p* ≤ 0.001). Simple comparison found that ST lesions were on average 34 − 13 = 21 mL/min/100 cm^3^ more increased than OM lesions. From logarithmic comparison, the increase was approximately four times higher in ST lesions than in OM lesions (logarithmic median 1.41). Note that these increases are from different baseline (non-infected) levels (see also the “Discussion” section).

## Discussion

Generally, infection is associated with inflammation and increased perfusion, increasing the possibility of leukocytes (and possibly antibiotics) reaching the infected site. That is, the tissue has a perfusion reserve which can be mobilized in such cases. In line with the non-quantitative studies mentioned in the introduction, i.e. [4, 5, 7, 9, 10], we found increased blood perfusion in the OM lesions (Table 3), as well as in the ST lesions (Table 4). However, as hypothesized, we found a quite clear distinction between the OM lesions and the ST lesions as the increase was significantly higher in the infected soft tissues than in the osteomyelitic lesions. Apparently, the perfusion reserve in osteomyelitic bone is less than the reserve in soft tissue infections.

This effect is illustrated by Fig. 4; especially the logarithmic differences (representing ratios) show a plain distinction between ST and OM lesions (Fig. 4, lower image). The typical perfusion increase was about 50% (a factor 1.5) in the OM lesions, but approximately a factor 6 in the ST lesions.

It should be noted, though, that these factors are calculated from different baseline levels for bone and soft tissue. In fact, Table 2 shows that the right side (infected) perfusion was on average ~40 mL/min/100 cm^3^ for both the OM and the ST lesions, i.e. about equal. It could be argued that the performance of the infected bone and soft tissue simply reflects the anatomy of the vascular tree (vascularization) in these two tissues, allowing low basal perfusion in ST, while the infected bone and soft tissue perform equally.

However, the full potential of bone perfusion was investigated in a study by Ashcroft et al. [11], who studied perfusion in human tibia fractures (age of the patients 20–28 years). Two weeks after fracture, blood flow was elevated up to 14 times normal in undisplaced fractures and 1.7 to 6.9 times normal in displaced fractures. Except for their lowest number, these ratios are comparable to our results for ST lesions, while being considerably higher than our ratios for OM lesions, strengthening our interpretation of the OM perfusion increase as being low.

The levels of perfusion found by us for non-infected marrow are comparable to the findings of a porcine study of vertebral bone [13]. A canine study [30] investigating both bone and muscle perfusion reported perfusion levels comparable to inactive pig data. Noteworthy, the canine study differentiated between yellow and red marrow, finding markedly less perfusion in yellow than in red marrow. Our pigs were juvenile, therefore being expected to contain red marrow in most bones. Studies of mature individuals (pigs or other) might find different bone perfusion levels in the tibial bone, for example, which will be expected to contain mostly yellow marrow.

The pathogenesis of haematogenous long bone osteomyelitis in children, juvenile pigs and other animals includes thrombosis of the capillary loops of the metaphysis and ischemic bone necrosis [3, 31, 32]. It is a general pathogenic assumption that the development of inflammation within the rigid confinement of bone, i.e. osteomyelitis, will cause increased intraosseous pressure and occlusion of blood vessels due to compression [32]. This compression force could relate to the absence of lymphatics in the bone, lymphatics being absent in the human bone [33], but occlusion of blood vessels due to compression has at least in one study in chickens been rejected, as newly formed blood vessels peripheral to the sequestrum/abscess were fully patent 8 days after experimental inoculation [31].

A low increase of blood perfusion in osteomyelitis could influence not only the healing process but also the effectiveness of antibiotics. In experimental pharmacokinetic studies in pigs with 5 days old implant-associated osteomyelitis, the antibiotic cefuroxime penetrated incompletely into the implant cavity; less incomplete penetration was noted for infected cancellous bone as opposed to infected subcutaneous tissue [34]. The authors speculated that differences in antibiotic penetration could be attributable to impaired blood flow.

Clinically, [^15^O]water is being used for perfusion studies, especially in the heart and the brain. The brief half-life (*T*_½_ = 2 min) of ^15^O limits the availability to PET scanning centres with an on-site cyclotron and necessitates efficient procedures and precise timing but gives the advantages of a low radiation dose (1.1 μSv/MBq for a standard 70-kg adult [35]) and the possibility of repeated scanning after a short waiting time. Quantitative unitless ratios, e.g. right/left as in the present study, can be determined without measurement of the blood input function, see, e.g. Ref. [11]. For quantitative measurements of perfusion in mL/min/100 cm^3^, the blood input function must be known. One possibility is arterial blood sampling (as used in the present study); however, this is clinically unattractive. Depending on what part of the body is scanned, it may be possible to determine the input function from a large blood pool within the scanner field-of-view, such as the heart, the aorta, or (with more difficulty) the carotis, especially if supplemented with a few blood samples [36–38].

Based on our findings, it seems that a suspected osteomyelitis diagnosis could be corroborated with [^15^O]water PET/CT, partly from the CT image and partly from elevated perfusion relative to the other side of the body. However, the investigated section will be limited to the scanner field-of-view, and as our data show, a negative PET result (perfusion not elevated) does not guarantee the absence of osteomyelitis. For these reasons, [^15^O]water PET/CT is not likely to become the technique of choice for diagnosing osteomyelitis.

[^15^O]water PET/CT may, however, provide information on the likelihood of antibiotic treatment success. We speculate that antibiotic treatment of osteomyelitis will have a higher success rate when perfusion is highly elevated than when it is only slightly elevated or even reduced. This speculation could be investigated in a human study with [^15^O]water PET/CT of osteomyelitis patients already decided for antibiotic treatment. Or better, an animal model study combining antibiotic and perfusion data could provide a more direct test and might even provide an algorithm to predict antibiotic effect from blood perfusion.

### Methodological considerations and limitations

Some methodological questions should be addressed in order to signify our results.

Using the left limb as a reference assumes that this limb could be considered healthy. We did occasionally see systemic spread to the lungs, and one OM lesion was found in the humerus of one pig [18–20]. However, no infection in the left hind limb was observed in any of the pigs on CT scans or during sections.

A second assumption was that the right and left limbs were comparable on the outset (i.e., no dominant side). Investigating femoral marrow and thigh muscles as seemingly uninfected tissue, we found only a small side difference in the muscles and of a size (about 11%) considerably smaller than the difference between infected and non-infected tissues.

Some VOIs of the OM lesions inevitably included small sequestra, i.e. necrotic bone, which by definition has no perfusion. *S. aureus* is a pyogenic bacterium, and histology indeed showed the OM lesions to be both necrotizing and suppurative and subacute with formation of a reparative inflammatory tissue [19, 20]. Accumulated neutrophils in abscess centres also represent necrotic tissue without perfusion as demonstrated in an abscess in pig no. 4 (Fig. 3). As the spatial resolution of the PET data was too poor to exclude small sequesters and suppuration, our finding that moving bone VOIs in different directions did not lead to higher perfusion values indicates that the chosen VOIs reported the average near-maximum perfusion, even of the heterogeneous OM lesions.

Regarding the age of the lesions, probably all of the peri-osseous ST infections, except the injection site abscess in pig no. 4 (Table 4), had developed by contiguous inoculation from the osteomyelitis; thus, the ST lesion may be considered younger (more acute) compared to the pathological subacute osteomyelitis [20]. Assuming that perfusion of acute infections are higher than perfusion of subacute infections (regardless of the lesion type), this would in itself result in a difference between ST and OM lesions. However, the possible variation in age of lesions is slight and unlikely to be the main problem; we still consider this to be the lower bone perfusion increase.

Finally, our method did not correct for dispersion of the arterial input function, which may result in underestimation of perfusion [39]; however, the simulation part of that study had about 11% underestimation as worst-case example (for a very high dispersion constant, *τ* = 10 s, and a high perfusion, *K*_1_ = 0.6 mL/min/g = 60 mL/min/100 g). For the quantification of OM perfusion, we consider this level of worst-case underestimation acceptable.

## Conclusions

Quantitative perfusion in and around osteomyelitic lesions can be determined with [^15^O]water dynamic PET/CT scan. Investigating pathological subacute haematogenous *S. aureus* osteomyelitic lesions in juvenile pigs, we have found that perfusion is elevated compared to corresponding non-infected bone, but the elevation is significantly lower (by a factor of about 4) than in soft tissue lesions induced by the same *S. aureus* inoculation. This seems to indicate less perfusion reserve in infected bones than in infected soft tissue, which could influence both successful therapy and healing.

## Additional file

Additional file 1: Supplementary figures and table. (DOC 93 kb)

## Abbreviations

CT: Computed tomography
OM: Osteomyelitic
PET: Positron emission tomography
ROI: Region of interest
ST: Soft tissue
VOI: Volume of interest
