# Supplementary material for: Blood perfusion in osteomyelitis studied with [15O]water PET in a juvenile porcine model
Source: EJNMMI Res. 2017 Jan 14;7:4. doi: 10.1186/s13550-016-0251-2 (PMC5237436; doi:10.1186/s13550-016-0251-2)
Supplement: Additional file 1: — Supplementary figures and table. (DOC 93 kb) [file 13550_2016_251_MOESM1_ESM.doc]

SUPPLEMENTARY MATERIAL


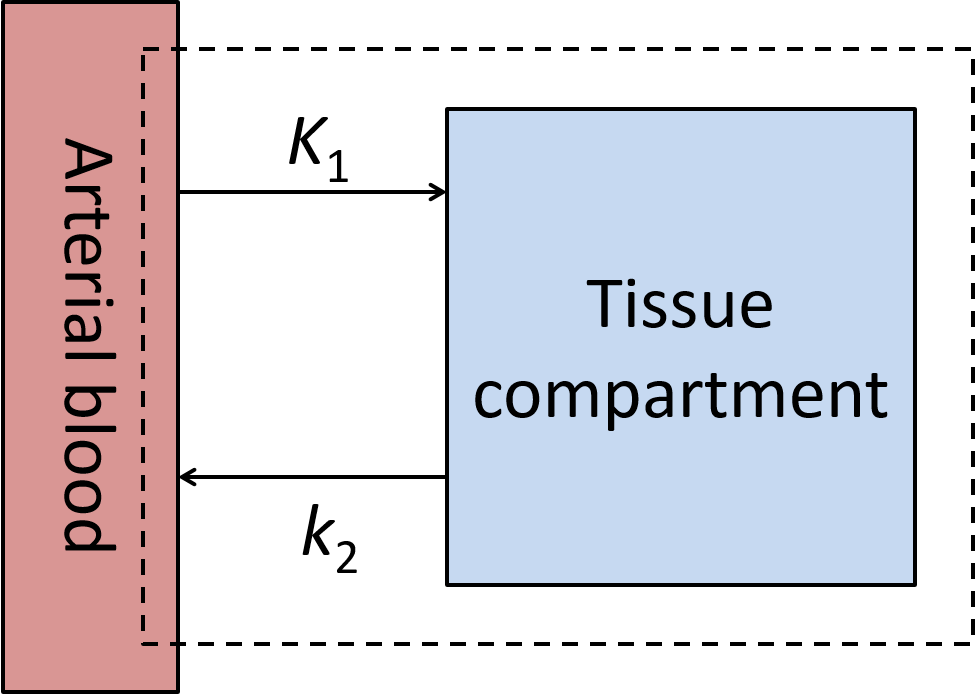


**Supplementary Figure 1**. Model used for kinetic modelling of [^15^O]water, comprising arterial input function and one tissue compartment. The measured PET signal is denoted by the dashed box; it is the sum of the tissue compartment and a fraction *V_a_* of the arterial input function. The parameter *K*_1_, with unit mL/min/100cm^3^, describes uptake and equals perfusion *F* multiplied by the extraction fraction *EF* for a single passage of the tracer: *K*_1_ = *EF* × *F*. For water as tracer, extraction is close to 100 %, and we assume *K*_1_ = *F*. The parameter *k*_2_ (unit min^-1^) is the fraction per time of tracer leaving the tissue compartment.


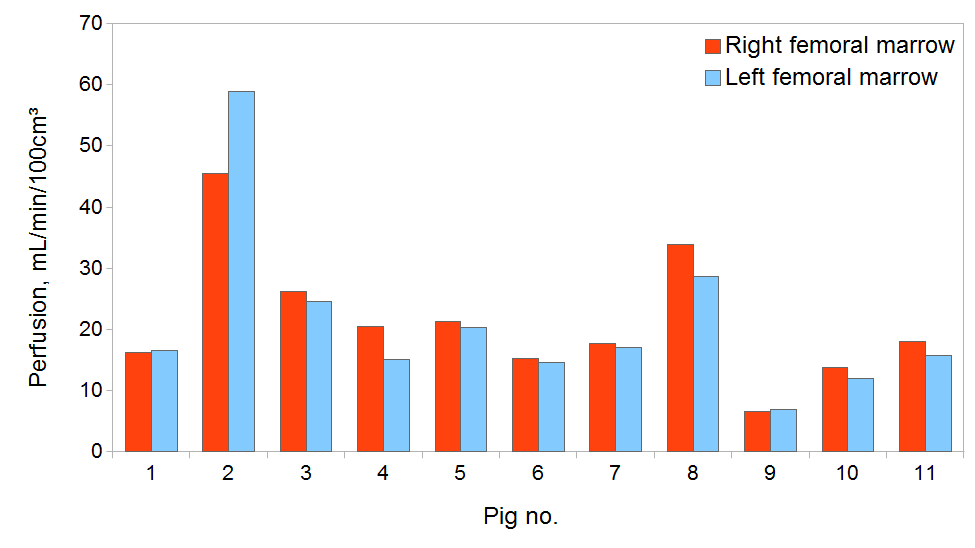


**Supplementary Figure 2**. Perfusion of the diaphysis medullar canal of the femur in the pigs, regions that always were without lesions. Apart from pig no. 2, which has an unexplained high perfusion of the medulla of the left (non-infected) limb, the diagram indicates only relatively small differences between the limbs.


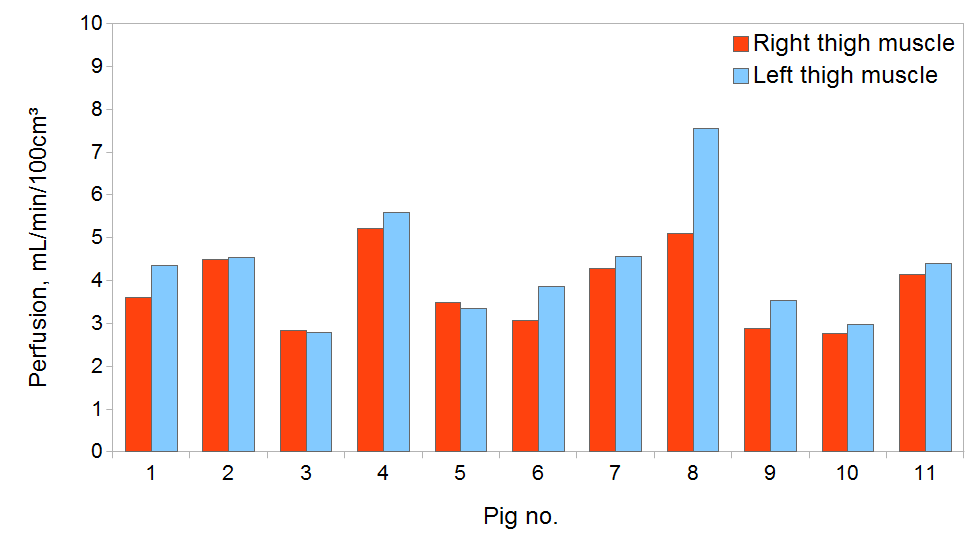


**Supplementary Figure 3**. Perfusion of thigh muscles in the pigs. Except for pig no. 8, the diagram indicates only small differences between the left (non-infected) and right (infected) limb. Overall, the level is low, but represents muscle perfusion at complete rest as the pigs were sedated.

**Supplementary Table 1**. Summary of statistics on differences between right and left limb

| **VOI location** | **Number of pairs** | **Difference type** | **mean (median) ± SD of differences** | **p-value** | **test *** |
| --- | --- | --- | --- | --- | --- |
| Medullary canal | 11 | simple | 0.4 (0.9) ± 5.0 mL/min/100cm^3^ | 0.1016 | Wilcoxon |
|  |  | logarithmic | 0.06 (0.05) ± 0.14 | 0.2073 | Paired t |
| Thigh muscles | 11 | simple | -0.5 (-0.3) ± 0.7 mL/min/100cm^3^ | 0.0098 | Wilcoxon |
|  |  | logarithmic | -0.11 (-0.07) ± 0.13 | 0.0169 | Paired t |
| OM lesions ^†^ | 17 | simple | 13 (12) ± 14mL/min/100cm^3^ | 0.0014 | Paired t |
|  |  | logarithmic | 0.38 (0.41) ± 0.37 | 0.0007 | Paired t |
| ST lesions ^‡^ | 8 | simple | 34 (34) ± 12 mL/min/100cm^3^ | 0.0001 | Paired t |
|  |  | logarithmic | 1.81 (1.74) ±0.50 | 0.0078 | Wilcoxon |

* If differences showed signs of non-normal distribution (Shapiro-Wilk W test) then Wilcoxon’s signed test was used for computation of p-value, otherwise Student’s paired t-test was used. All reported *p*-values are two-sided.

**^†^** Only long bones (not patella); primary data in Table 3 of main paper.

^‡^ Primary data in Table 4 of main paper.
